# An AI-Assisted Cognitive Engagement Mobile App for Older Adults: Development and Mixed Methods Usability Study

**DOI:** 10.2196/103986

**Published:** 2026-09-10

**Authors:** Hojin Shin, Minjun Shin

**Affiliations:** 1 Hamilton Southeastern High School, Fishers, IN, United States; 2 Department of Biology, Indiana University Bloomington, Biology Building 1001 East Third Street, Bloomington, IN, 47405-7005, United States, 1 812-855-7323

**Keywords:** AI-assisted cognitive engagement, mobile health apps, facial expression recognition, older adults, human-centered AI

## Abstract

**Background:**

Alzheimer disease and age-related cognitive decline reduce memory engagement and limit caregiver insight, creating a need for accessible tools that support everyday cognitive activity in older adults. Although AI holds promise for personalized cognitive support, few AI-based apps have been developed and evaluated for memory engagement in this population, and fewer incorporate on-device emotional analysis with privacy-preserving design.

**Objective:**

This study developed and evaluated RecallLive, an AI-assisted mobile app supporting structured memory interactions for older adults, and examined its usability, engagement, perceived usefulness, and behavioral intention, guided by the Technology Acceptance Model.

**Methods:**

RecallLive integrates metadata-based photo clustering to generate memory videos, an on-device convolutional neural network that classifies frame-level emotional responses, and a large language model (LLM)–based module that converts these outputs into caregiver summaries. A sequential 2-phase mixed methods design was used. In phase 1, 202 US adults aged 65 years or older viewed a structured demonstration and completed a survey measuring ease of use, engagement, design clarity, perceived usefulness, and intention to use. In phase 2, 10 participants completed a hands-on session followed by semistructured interviews analyzed thematically. Quantitative analyses were conducted using Python (v.3.11; Python Software Foundation) and included internal consistency estimates; 2-tailed, 1-sample *t* tests against the scale midpoint; and simple linear regression.

**Results:**

All subscales showed strong reliability (Cronbach *α*=0.86-0.92). Ease of use (mean 3.77, SD 0.78) and engagement (mean 4.09, SD 0.67) significantly exceeded the scale midpoint (*t*_201_=14.00 and *t*_201_=23.19; both *P*<.001), and perceived usefulness was also rated highly (mean 4.06, SD 0.82). Perceived usefulness was strongly associated with the stated intention to use or recommend the app (β=.796; *R*²=0.634; *F*_1,200_=346.18; *P*<.001). Phase 2 complemented these results, showing clear navigation and interpretable feedback.

**Conclusions:**

RecallLive was perceived as usable, engaging, and useful, with perceived usefulness strongly associated with adoption intention. In addition to efficiency, emotional relevance shaped engagement, particularly among participants with caregiving experience or personal connections to dementia. Future studies should use larger samples, longer direct-use periods, and longitudinal and clinical-integration designs to evaluate sustained use and cognitive outcomes.

## Introduction

### Background

Population aging has intensified the need for accessible tools that support cognitive health and everyday functioning among older adults. Cognitive decline, including memory impairment and reduced attentional capacity, affects not only clinical populations but also older adults seeking to maintain independence and quality of life [1,2]. Digital interventions for older adults have become increasingly common, yet many continue to center on specific support functions, such as reminders, monitoring, and structured cognitive exercises, while tailoring them to individual needs and sustaining active, meaningful engagement remain recognized design challenges [3-5]. Emerging advances in AI offer new opportunities to address these challenges; in particular, the integration of machine learning with personal data allows systems to move beyond generic content delivery and toward individualized, context-aware interaction [6,7]. However, evidence remains limited regarding AI-enabled systems that integrate personalized memory interaction, emotional-response analysis, and caregiver-oriented interpretation within a single app [4,6,7], and many existing apps in this domain focus on prediction or automation rather than interaction with personally meaningful content [8]. This leaves a gap for systems that actively engage users while remaining interpretable and easy to use, supporting ongoing cognitive engagement and providing meaningful feedback for both users and caregivers [4,9].

### Rationale

To address the gap, this work presents the development and evaluation of RecallLive, an AI-assisted mobile system designed to support cognitive engagement through structured interaction with personal memories. Within the app, personal photos are clustered into meaningful events using temporal and spatial metadata and are transformed into structured memory videos; during playback, the system analyzes facial expressions in real time through the smartphone camera to construct a continuous emotion timeline. These responses are then aggregated into interpretable summaries presented through a caregiver-facing dashboard, creating a feedback loop between memory exposure and emotional engagement.

Because the potential value of this integrated system depends not only on its technical operation but also on whether older adults perceive it as usable, engaging, and useful, a formative evaluation of its usability, engagement, perceived usefulness, and adoption potential is warranted. The Technology Acceptance Model (TAM) identifies perceived usefulness and ease of use as important determinants of behavioral intention [10], making it an appropriate framework for examining RecallLive’s adoption potential. Engagement was included as a complementary experiential construct, as RecallLive is intended to encourage meaningful interaction with personal memories in addition to the efficient completion of functional tasks.

### Study Objectives

Accordingly, this study aimed to provide a comprehensive understanding of how older adults experience AI-assisted cognitive engagement systems and whether such systems can support meaningful interaction in everyday contexts. It was guided by the following research question: Is an AI-assisted mobile app integrating metadata-based clustering, real-time facial expression analysis, and language-model summarization perceived as usable, engaging, and useful by older adults? In addressing this question, the study evaluates whether the RecallLive system is perceived as usable, engaging, and valuable when older adults are introduced to its core functions and when they directly interact with the app. We hypothesized that an AI-assisted system integrating visual memory interaction and emotional response analysis would foster high levels of usability, engagement, perceived usefulness, and intention to use among older adults. Based on these objectives, the following hypotheses are proposed:

- H1a: Older adults will report high perceived usability of the RecallLive system.
- H1b: Older adults will report high engagement with the RecallLive system.
- H2: Higher perceived usefulness will be positively associated with the combined intention to use and recommend the app.

To evaluate the system across both initial perceptions and direct use, we used a sequential 2-phase mixed methods design: phase 1 provided a large-scale, perception-based evaluation in which older adults viewed a standardized demonstration and completed a structured survey, and phase 2 examined direct interaction through a hands-on usability session followed by semistructured qualitative interviews. Detailed procedures for both phases are described in the *Methods* section.

## Methods

### System Design and Architecture

#### Overview

**Figure 1.**
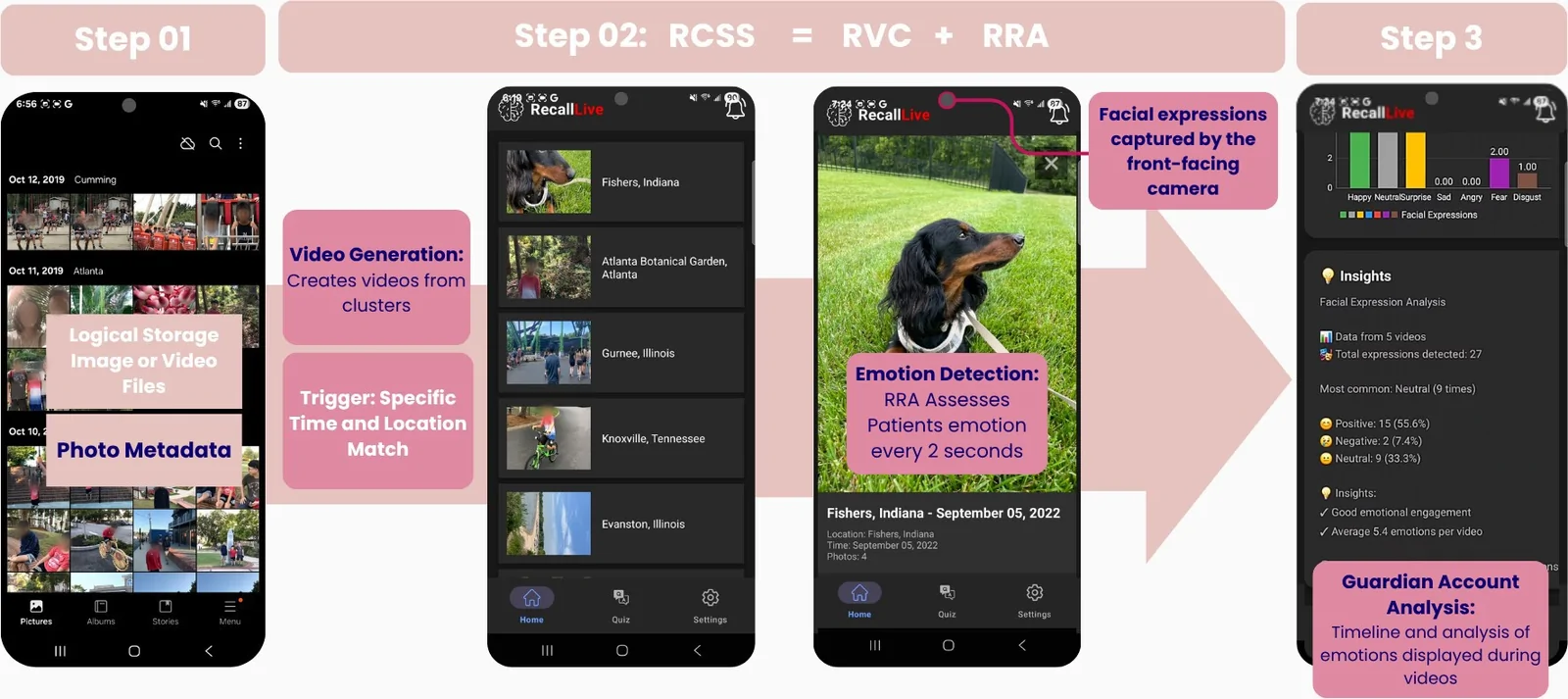
Workflow of the RecallLive Cognitive Support System (RCSS). RRA: ReLive Response Analyzer; RRG: ReLive Report Generator; RVC: ReLive Visual Composer.

**Figure 2.**
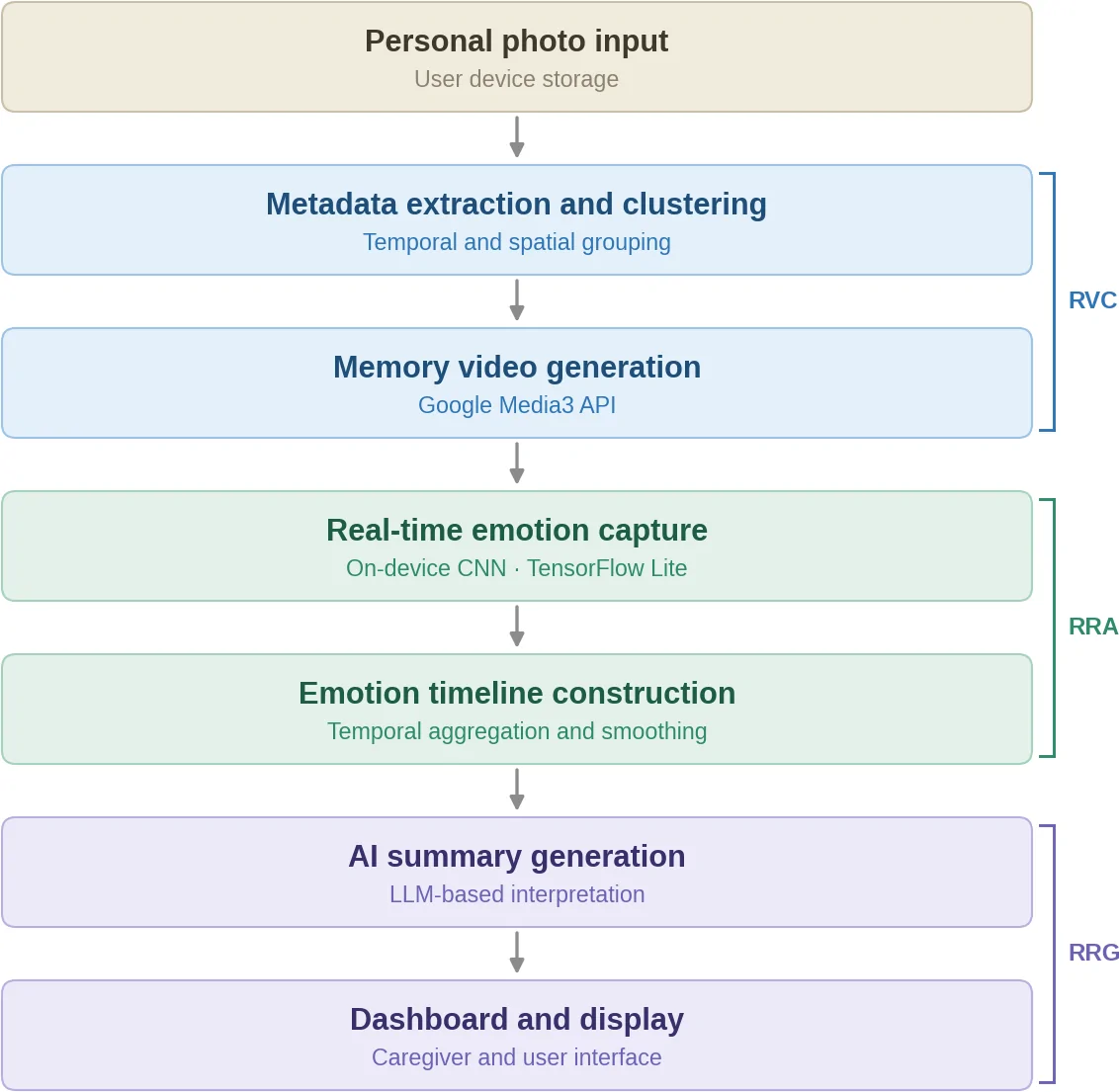
End-to-end processing pipeline of the RecallLive Cognitive Support System (RCSS). CNN: convolutional neural network; LLM: large language model; RRA: ReLive Response Analyzer; RRG: ReLive Report Generator; RVC: ReLive Visual Composer.

The RecallLive system [11] was designed and implemented as an end-to-end mobile architecture that integrates personal data processing, real-time affective computing, and AI-driven interpretive feedback within a unified cognitive support environment. The system operates through a tightly integrated workflow that connects memory construction, emotional analysis, and interpretive reporting (Figure 1). Unlike conventional mobile apps that treat media consumption, analytics, and reporting as separate processes, RecallLive operationalizes these components as a continuous pipeline. This integrated design reflects the project’s core objective of moving beyond static memory aids and enabling dynamic, data-driven interaction among users, their memories, and caregivers. The logical processing pipeline of the RecallLive Cognitive Support System (RCSS; Figure 2) illustrates the sequential data flow across its 3 subsystems. The pipeline originates with personal photo input and proceeds through metadata-driven clustering and video generation within the ReLive Visual Composer (RVC), real-time emotion capture and timeline construction within the ReLive Response Analyzer (RRA), and, finally, large language model (LLM)–based summary generation and dashboard visualization within the ReLive Report Generator (RRG).

#### RVC: Structured Memory Construction

The RVC serves as the system’s input processing and memory structuring module. Its primary function is to convert unstructured photo collections into coherent narrative units that can facilitate reminiscence and cognitive stimulation. The system extracts EXIF metadata from each image, including timestamp and GPS location, which are then used to implement rule-based clustering algorithms. Temporal clustering groups images captured within defined time windows (eg, same day or event period), while spatial clustering groups images taken in geographic proximity. This dual-layer clustering strategy enables the system to reconstruct meaningful life events, such as family gatherings or travel experiences, without requiring manual user input. Once clustered, images are transformed into short-form memory videos using the Google Media3 API [12]. The system enforces chronological ordering, applies minimal transitions to maintain visual continuity, and constrains video duration to reduce cognitive overload—an important design consideration for older adults. As shown in the system design materials, this process is intentionally optimized for clarity and familiarity rather than visual complexity. Importantly, this module reflects a conceptual shift from static photo viewing to structured memory storytelling, which prior research suggests enhances emotional recall and engagement. By presenting memories as temporally organized narratives, the RVC establishes the foundation for downstream emotional analysis.

#### RRA: On-Device Affective Computing

The RRA constitutes the system’s core AI component, responsible for capturing and interpreting users’ emotional responses during memory interaction. During video playback, the smartphone’s front-facing camera captures continuous image frames. These frames are processed in real time using a convolutional neural network deployed via TensorFlow Lite, enabling on-device inference. The facial expression analysis draws on established facial behavior coding and facial expression recognition frameworks and resources [13,14]. The model classifies each frame into 1 of 7 emotion categories: happiness, sadness, surprise, anger, fear, disgust, and neutrality. Each frame produces a probability distribution across these emotional categories. Rather than relying on discrete classification, the system aggregates these probabilistic outputs over time to construct a continuous emotional timeline for each video. Temporal smoothing techniques are applied to reduce noise and ensure stability in emotion trajectories. A key design decision is the use of fully on-device processing, which eliminates the need to transmit facial data to external servers, addressing critical privacy concerns associated with facial recognition technologies while also improving latency and responsiveness. By design, no raw facial images are stored or transmitted; only aggregated emotion metrics are retained. Beyond individual videos, the system performs cross-video aggregation to identify broader engagement patterns, including detecting trends in positive versus negative emotional responses and identifying which memory clusters elicit stronger emotional reactions. This transforms the system from a simple emotion detector into a longitudinal affective analytics tool.

#### RRG: AI-Based Interpretability Layer

While the RRA produces rich quantitative emotion data, such outputs are difficult for nontechnical users to interpret. The RRG addresses this gap by transforming structured emotional data into natural language summaries that are accessible and meaningful in everyday contexts. The system receives aggregated emotion distributions, temporal patterns derived from emotion timelines, and metadata associated with memory clusters and transmits these structured inputs to an LLM through an API. The model then generates descriptive summaries of user engagement, translating numerical patterns into interpretable insights. For instance, the system may indicate that positive engagement is more prominent during recent family-related memories, while more neutral or reflective responses occur when viewing older photo sets. This transformation from quantitative outputs to narrative interpretation enables caregivers to understand emotional responses without requiring technical expertise. These summaries are integrated into a visual analytics interface that presents emotion timelines, aggregate distributions, and key insights, allowing caregivers to monitor emotional well-being and identify memory content that elicits meaningful engagement.

#### Data Architecture and System Integration

The RecallLive system is supported by a cloud-based backend architecture built on Firebase services, enabling scalable data management and real-time synchronization across devices. The design of the data architecture reflects a hybrid approach that strategically combines on-device processing with cloud-based storage and communication, ensuring both privacy preservation and system responsiveness. At the core of the system, the Firebase Realtime Database is used to manage dynamic user-related data, including user profiles and aggregated emotional response outputs [15]. This component enables continuous synchronization between the mobile app and the caregiver interface, allowing emotional analytics to be updated and accessed in real time. Such functionality is essential for supporting remote monitoring scenarios in which caregivers rely on up-to-date information to interpret user engagement patterns. In parallel, the Firebase Firestore is used to store structured data associated with system-generated artifacts, including metadata related to photo clusters, video identifiers, and engagement summaries. Although the Realtime Database prioritizes rapid synchronization, Firestore provides a flexible and scalable solution for organizing semistructured data, allowing the system to efficiently manage relationships between memory clusters and their corresponding analytical outputs.

Firebase Storage serves as the primary repository for media content, including the memory videos generated by the ReLive Visual Composer [16]. The architectural separation of media storage from structured data management ensures efficient handling of large files while maintaining fast access to metadata and analytical results, enabling RecallLive to balance privacy, performance, and scalability. Sensitive operations, such as facial expression analysis, are performed entirely on-device, eliminating the need to transmit raw visual data and thereby reducing privacy risks. At the same time, cloud-based services support data persistence, cross-device accessibility, and caregiver integration. This hybrid design is particularly well-suited for real-world deployment, where both individual users and external stakeholders interact with the system in distributed environments.

### Study Design

A sequential mixed methods design was used to evaluate the RecallLive system. The first phase examined user perceptions following a controlled demonstration, while the second phase investigated direct interaction through hands-on use. This approach aligns with recent formative studies that combine quantitative and qualitative evidence to evaluate user perceptions and experiences [17,18]. Participants were older adult users and possessed varying levels of familiarity with mobile technologies. The evaluation was structured into 2 phases to better integrate large-scale perceptual data with in-depth experiential insights.

### Phase 1 Participants and Procedures

Phase 1 participants were required to meet these inclusion criteria: (1) age 65 years or older, (2) current residence in the United States, (3) ability to provide electronic informed consent, and (4) ability to view and hear the audiovisual demonstration and complete the online survey using an internet-connected device. Participants were recruited nationally through Connect (CloudResearch), an online research platform, allowing for broad access to a geographically diverse sample. The survey was voluntary and closed, accessible only to panel members who received the study invitation, and it was not publicly advertised. Each panel account could submit the survey only once, which prevented duplicate responses from the same participant; no cookie or IP address checks were separately applied. Geographic information beyond US residence was not collected; therefore, participants’ states and cities of residence were unavailable. Specific medical diagnoses and hearing, vision, or motor impairments were neither used as exclusion criteria nor collected as participant characteristics, and no cognitive screening or assessment was administered.

To ensure consistency in system exposure, all participants were presented with a structured demonstration video that introduced the key functionalities of RecallLive, including metadata-based photo clustering, memory video generation, real-time facial emotion analysis, and AI-generated summaries for caregiver interpretation. Following the demonstration, participants completed a structured questionnaire comprising 16 evaluation items and 7 sociodemographic items presented over 2 screens, with sociodemographic items on one page and all 16 evaluation items on a second page. The evaluation items assessed multiple dimensions of user perception, namely ease of use, engagement, design clarity, perceived usefulness, and intention to use or recommend the system, and the sociodemographic items collected age, gender, ethnicity, marital status, education, employment status, and total household income. The order of the 16 evaluation items was randomized for each participant, and no adaptive questioning was used. All items required a response before submission, and participants could navigate back to review and change their answers before submitting. The measures were selected to reflect both functional usability and experiential engagement, recognizing that the system is designed not only to be usable but also to foster meaningful interaction with personal memories. This phase provided a large-scale assessment of how the system is perceived prior to direct interaction, offering insight into initial acceptance and perceived value.

### Phase 1 Measures

All 16 items across the 5 dimensions were rated on a 5-point Likert scale (1=strongly disagree; 5=strongly agree) and were adapted from prior studies: 4 items assessed ease of use and 3 assessed perceived usefulness, with 1 intention-to-use item adapted from Davis [10]; 4 items assessed engagement based on O’Brien et al [19]; 3 items assessed design and accessibility based on Gomez-Hernandez et al [9]; and 1 recommendation item was adapted from Stoyanov et al [20]. Composite scores were calculated by averaging the items within each dimension, with higher scores indicating more favorable perceptions and 3 representing a neutral response. The intention-to-use item (“I would be willing to use this app regularly”) and the recommendation item (“I would recommend this app to other older adults or families”) were averaged into a single 2-item intention or recommendation score, reflecting a broader adoption-oriented response.

### Phase 2 Participants and Procedures

Phase 2 participants were required to meet these inclusion criteria: (1) completion of phase 1, (2) ownership of a compatible Android smartphone, (3) ability to install and use the APK (Android Package Kit)-based app, and (4) willingness to complete a hands-on usability session and a semistructured interview. Following phase 1, an invitation describing these criteria was sent to all phase 1 respondents, and volunteer sampling within these predefined eligibility criteria was used [21]. All respondents who volunteered and met the criteria were enrolled. A target of approximately 10 participants was informed by empirical usability research indicating that 10 users can identify at least 80% of usability problems [22]. The recruitment, scheduling, hands-on sessions, and interviews were completed within 1 week after participants completed phase 1. No additional medical, cognitive status, or sensory exclusion criteria were applied, and no cognitive assessment was administered in this phase.

Although phase 1 relied on standardized video-based exposure, phase 2 allowed participants to engage with their own photo collections, thereby introducing personally meaningful content into the interaction process. During the approximately 30-minute session, participants interacted with the 4 interaction features: they generated memory videos through the system’s metadata-based photo clustering, viewed the resulting videos while the front-facing camera performed real-time facial expression analysis, completed the interactive memory quiz, and reviewed the engagement summary. This enabled the system to construct emotion timelines and produce engagement summaries based on actual user responses. Afterward, a semistructured interview lasting an average of 30 minutes was conducted via videoconference to capture detailed reflections on usability, navigation, emotional engagement, clarity of feedback, and overall experience.

The interview guide (Multimedia Appendix 1) was developed to align with the 5 dimensions measured in the phase 1 survey, with additional questions comparing hands-on use with the phase 1 demonstration and eliciting open-ended feedback. The guide was iteratively developed by the research team and reviewed by 2 external scholars with expertise in qualitative research, whose feedback informed revisions before the guide was finalized. Interviews were audio-recorded with participants’ consent; after transcript verification, the audio files were permanently deleted. These interviews provided insight into how users interpret and respond to the system when interacting with it directly [23].

### Data Analysis

Quantitative data collected in phase 1 were analyzed using descriptive statistics to summarize overall trends. Internal consistency was assessed using Cronbach *α* [24]. One-sample, 2-tailed *t* tests compared the ease-of-use and engagement composite means with the neutral midpoint of 3.0 to evaluate H1a and H1b. Simple ordinary least squares (OLS) linear regression was used to examine the association between perceived usefulness and the 2-item intention or recommendation score. All quantitative analyses were performed using Python (v.3.11; Python Software Foundation). Qualitative data from phase 2 were transcribed verbatim and analyzed using thematic analysis, following established procedures that emphasize iterative coding and theme development from qualitative data [25]. Transcripts were read repeatedly for familiarization and coded, and codes were grouped into candidate themes organized around the interview domains of usability, navigation, emotional engagement, clarity of feedback, and overall experience. Themes were then reviewed and refined against the full dataset to ensure they accurately represented participant responses. The integration of these 2 phases enabled a comprehensive evaluation of the RecallLive system by combining broader perception-based findings with detailed experiential insights derived from real-world interaction, with phase 2 themes compared with the phase 1 quantitative results during interpretation.

### Ethical Considerations

This study was reviewed and approved before data collection by the regional Scientific Review Committee of the International Science and Engineering Fair and the school-based Institutional Review Board of Hamilton Southeastern High School, Indiana, USA (approval date: February 2026) and was classified as minimal risk. No approval number was assigned; under this review process, approval was documented through signed and dated review forms rather than by an approval number. All participants were adults aged 65 years or older, and participants in both phase 1 and phase 2 completed an electronic informed consent after receiving information about the study purpose, procedures, potential risks, privacy protections, and their rights as research participants. Participation was voluntary, and participants could withdraw at any time without penalty. Phase 1 data collection was conducted on March 20, 2026, and phase 2 interviews were conducted from March 20 to March 26, 2026. No minors, medical procedures, diagnostic assessments, treatment, or medical records were involved. Survey responses were collected without names or directly identifying information. All research data were analyzed in deidentified form. During phase 2, facial expression analysis was performed on-device; raw facial images or videos from the front-facing camera were not stored or transmitted, and only aggregated engagement metrics were retained for analysis. Phase 2 interview recordings were permanently deleted after transcript verification. Participants received US $1.50 for participation in phase 1 and US $50 for participation in phase 2.

## Results

### Results of Study Phase 1

#### Sample Characteristics

Of the 230 panel members who opened the survey, 202 (participation rate 87.8%) provided consent and completed the questionnaire in full; only completed questionnaires were analyzed, and no responses were excluded for atypical completion times. Participant age ranged from 65 to 84 (mean 70.51, SD 4.40) years. The sample included 78 (38.6%) male participants, 122 (60.4%) female participants, and 2 identifying otherwise (1.0%). The sample was predominantly Caucasian (n=183, 90.6%), while 19 (9.4%) identified with other racial or ethnic groups. Marital status was nearly evenly divided between participants who were married (n=102, 50.5%) and those who were not (n=100, 49.5%). More than half held a bachelor’s or graduate degree (n=114, 56.4%), whereas the remainder had completed high school or an associate degree. Most (n=129, 63.9%) participants were retired, while 63 (31.2%) were employed. Annual household income was distributed as follows: ≤US $34,999, 42 (20.8%); US $35,000-US $49,999, 45 (22.3%); US $50,000-US $74,999, 41 (20.3%); US $75,000-US $99,999, 29 (14.4%); US $100,000-US $149,999, 33 (16.3%); and ≥US $150,000, 12 (5.9%).

#### Findings

Overall, participants responded positively to the RecallLive system across all dimensions (Table 1). The highest-rated item was “The app looked interesting” (mean 4.37, SD 0.72), followed by items reflecting perceived benefits for older adults and appropriateness for senior users. At the other end, intention to use the app received comparatively low ratings but remained above the midpoint of the scale, indicating a generally favorable but cautious adoption tendency.

**Table 1.** Item-level descriptive statistics (N=202).

Survey item	Mean (SD)
Section A: ease of use	
The app was easy to navigate.	3.77 (0.81)
The instructions were clear and understandable.	3.73 (0.97)
I was able to complete tasks without confusion.	3.75 (0.92)
The buttons and text were easy to read.	3.81 (0.79)
Section B: engagement	
The photo or video features were engaging.	4.07 (0.73)
The app encouraged me to reflect on memories.	4.00 (0.87)
I found the experience enjoyable.	3.90 (0.88)
The app looked interesting.	4.37 (0.72)
Section C: design and accessibility	
The layout was simple and looked good.	3.82 (0.83)
The visual design of the app felt appealing.	4.01 (0.75)
The visual layout was comfortable for me.	3.87 (0.82)
Section D: perceived usefulness	
This app would be helpful for memory engagement in daily life.	4.01 (0.85)
Using this app feels beneficial for older adults.	4.10 (0.88)
The app felt appropriate for senior users.	4.07 (0.94)
Section E: intention or recommendation	
I would be willing to use this app regularly (daily or weekly).	3.65 (0.96)
I would recommend this app to other older adults or families.	3.79 (0.97)

As shown in Table 2, all 5 dimensions demonstrated strong internal consistency. Engagement received the highest composite mean (mean 4.09, SD 0.67, *α*=.862), followed by perceived usefulness (mean 4.06, SD 0.82, *α*=.909). Design and accessibility was also rated positively (mean 3.90, SD 0.74, *α*=.917), while ease of use showed a moderately high mean (mean 3.77, SD 0.78, *α*=.919). Intention and recommendation produced the lowest composite mean (mean 3.72, SD 0.90, *α*=.863). All told, the full 16-item scale demonstrated strong internal consistency (mean 3.92, SD 0.68, *α*=.960).

**Table 2.** Composite subscale scores and Cronbach α (N=202).

Subscale	Items, n	Mean (SD)	α
Ease of use	4	3.77 (0.78)	.919
Engagement	4	4.09 (0.67)	.862
Design and accessibility	3	3.90 (0.74)	.917
Perceived usefulness	3	4.06 (0.82)	.909
Intention or recommendation	2	3.72 (0.90)	.863
Full scale (all 16 items)	16	3.92 (0.68)	.960

The results indicated that ease of use (mean 3.77, SD 0.78, *t*_201_=14.00; *P*<.001) and engagement (mean 4.09, SD 0.67, *t*200=23.19; *P*<.001) were significantly higher than the midpoint. These findings provide statistical support for H1a and H1b, confirming that older adults reported positive levels of usability and engagement when evaluating the RecallLive system. Taken together, both descriptive and inferential results indicate that participants perceived the RecallLive system as usable, engaging, and beneficial; moreover, the relatively high levels of engagement and perceived usefulness suggest that participants responded positively not only to the functional aspects of the system but also to the experiential dimension of interacting with personalized memory content.

The results of a simple OLS regression analysis showed a strong positive association between perceived usefulness and intention or recommendation score (*B*=0.880, SE =0.047, *β*=.796, *t*=18.606; *P*<.001), and the model explained a substantial proportion of variance in intention or recommendation score (*R*²=0.634; Table 3), indicating strong explanatory power. These findings support H2, as participants who reported higher perceived usefulness also tended to report stronger intentions to use or recommend the app. While the descriptive results indicate generally positive perceptions of the system, the regression results further indicate that perceived usefulness was strongly associated with the intention or recommendation score.

**Table 3.** Hypothesis testing results: 1-sample, 2-tailed *t* tests (H1) and simple linear regression (H2).

Hypothesis	*B*	SE	*β*	*t* test (df)	*P* value	*R*²	Result
H1a^a^	—^b^	—	—	14.00 (200)	<.001	—	Supported
H1b^c^	—	—	—	23.19 (200)	<.001	—	Supported
H2^d^	0.880	0.047	.796	18.606 (200)	<.001	0.634	Supported

a H1a: Older adults will report high perceived usability of the RecallLive system.

b Not applicable.

c H1b: Older adults will report high engagement with the RecallLive system.

d H2: Higher perceived usefulness will be positively associated with the combined intention to use and recommend the app.

### Results of Study Phase 2

#### Overview

To further examine how older adults interact with the system during direct use, all 202 phase 1 participants were invited to undertake hands-on interaction with the RecallLive app on their smartphones. Of the 12 who expressed interest in participating in phase 2, two were unable to proceed because the app could not be installed on their Android devices due to device compatibility limitations. The final phase 2 sample comprised 10 participants aged 65 to 76 (mean 69.9, SD 3.67) years; 6 were female and 4 were male. Five identified as African American, 2 as Caucasian, 2 as Asian, and 1 as Hispanic. Seven participants were married, and 9 were retired; household income ranged from ≤US $34,999 to US $100,000-US $149,999. Educational attainment ranged from less than high school to graduate education. Participants reported varying levels of prior experience with mobile technology. The findings from the hands-on sessions and post-use interviews are organized below by the 3 identified themes, namely ease of navigation, feature clarity, and interaction flow and user comfort; illustrative quotations are presented in Table 4.

**Table 4.** Illustrative participant quotations.

Theme	Quotation
Ease of navigation	
Overall usability	“The controls were very simple, so I didn’t have any trouble using it. It was easy to understand and operate overall.” (P5, male, 65)
Initial onboarding friction	“It was fairly straightforward, but I did run into a couple moments where I wasn’t sure what to do next. I had to slow down and look around a bit more than I expected. Once I figured it out, it worked fine.” (P8, female, 75)
Account linking	“The initial sign up from both patient and guardian and connecting the two accounts was a bit confusing.” (P7, male, 68)
Growing confidence	“It was okay overall, but I had to go back once or twice to make sure I did things right. It wasn’t difficult, just needed attention. After that, it felt easier.” (P10, female, 66)
Feature clarity	
Photo clustering and memory videos	“The pictures were super cool. They were organized into places where the pictures were taken. So I did like that a lot.” (P4, female, 76)
Emotional resonance of videos	“I actually enjoyed watching the little videos. It kind of reminded me of old times, so I found myself paying attention more than I expected.” (P6, female, 72)
Memory quiz	“I loved the post quiz section and reading the summary.” (P9, male, 70)
Engagement summary	“The summary was definitely the standout feature for me. That part grabbed my attention the most and gave the app a really unique value compared to the usual stuff.” (P7, male, 68)
Interaction-level accessibility barrier	“The one thing I did notice was, when I was taking the test on my phone, I had to scroll up to enter... an older person with dementia might have difficulties with that.” (P3, female, 67)
Interaction flow and user comfort	
Repurposing personal photos	“Usually, photos just sit there taking up storage, but this felt like a way to actually use them in a beneficial way, especially for memory and mental engagement.” (P5, male, 65)
Improvement suggestions	“Maybe make the steps a little clearer in the beginning. A short guide would help people like me.” (P6, female, 72) “It would be nice to expand accessibility features—for example, audio-based interaction, incorporating familiar voices (like family members)” (P5, male, 65)
Caregiving connection	“My mother had dementia before she passed away, and I think she would have enjoyed it.” (P1, male, 71)

#### Ease of Navigation

Participants described the app as intuitive and logically structured. Overall, they perceived the step-by-step flow of the interface as accessible despite varying levels of technology familiarity; 9 of 10 participants progressed successfully through the system without external assistance. Four participants noted that the visual cues and screen layout reduced the need for prior instruction, describing the experience as self-guiding. One remarked that moving between sections felt natural, requiring little deliberate effort to orient within the app. Those with limited prior smartphone experience (3/10) showed some initial hesitation at the onboarding stage; as P6 (female, 72) described, “At first I was a little unsure where to go, but once I tapped around a bit, it got easier.” However, they reported increased confidence as the interaction progressed. The account-linking step between the caregiver and patient roles was cited by 4 of 10 participants as a source of initial confusion; P2 (female, 69) noted, “It was relatively user-friendly, except when I had to [do] that guardian and patient thing, they didn’t connect...at the first trial.” All participants were able to complete the full interaction sequence, suggesting that the navigation design was manageable for participants in this sample.

#### Feature Clarity

Seven of 10 participants highlighted the photo clustering and video generation feature as the app’s most salient and meaningful component. They also expressed appreciation for the automated organization of personal photos into temporally coherent narrative sequences, valuing its low user burden: as P1 (male, 71) remarked, “I didn’t have to go through and make a bunch of choices … a couple minutes later, they were all there.” Four of 10 described the experience of watching their own memory videos as emotionally resonant, with the videos prompting spontaneous recall of associated events and people. As one participant conveyed, “they reminded me of the people I shared those moments with. That helped bring back memories I might have otherwise forgotten” (P5, male, 65). Six of 10 also responded positively to the memory quiz feature, describing it as engaging (“the quiz was kind of fun,” P2). Participant accounts further supported the app’s interactive elements as promoting active cognitive engagement rather than passive viewing, and indicated the engagement summary to be understood and well received, with suggested refinements centering on accessibility (eg, audio-based interaction and clearer initial guidance).

#### Interaction Flow and User Comfort

Participants reported a comfortable and positive experience across the full session. They perceived the sequential structure of the app to be coherent, describing the transitions between features as smooth and predictable, with no notable instances of confusion when progressing from video viewing to quiz interaction to summary review. Regarding the visual design, 7 of 10 participants described the interface as simple, clean, or uncluttered, perceiving its simplicity as well suited to the app’s intended users and as contributing to a low-stress interaction experience. As P10 (female, 66) noted, “It looked nice and clean. I didn’t feel overwhelmed. The design felt comfortable to use.” Notably, the hands-on sessions revealed an additional pattern not prominent in the initial phase: participants with family caregiving experience or personal connections to Alzheimer disease expressed heightened emotional engagement during the memory video feature, describing the experience as personally meaningful and perceiving strong value in the system for those affected by cognitive decline. As P1 (male, 71), whose mother had lived with dementia, reflected, “I think she would have enjoyed it.” Collectively, the phase 2 findings corroborate and enrich the quantitative phase 1 results, confirming that RecallLive supports accessible navigation, meaningful feature interaction, and emotionally engaged use among older adults across diverse technology backgrounds.

## Discussion

### Principal Findings

The results suggest that the RecallLive system was perceived as both usable and meaningful by older adults in this initial evaluation. Participants reported positive evaluations across usability, engagement, and usefulness, and regression analysis showed that perceived usefulness was strongly associated with stated intention to use or recommend the app. This association is consistent with prior research linking perceived usefulness with behavioral intention [10,26]. However, recent evidence suggests that older adults vary substantially in their readiness to adopt digital health technologies. One identified subgroup perceived these technologies as useful but reported low self-efficacy and low perceived ease of use, indicating that favorable perceptions of usefulness may coexist with a need for operational support [27].

The qualitative findings suggest that usefulness alone may not fully explain the initial user experience. Participants described emotional reactions while viewing memory videos, indicating that engagement appeared to be related to personal relevance and reflection. Participant accounts indicated that seeing their own photos in sequence helped them recall past events and maintain attention longer than expected. This highlights an important point for system design, in that apps for older adults should not focus only on efficiency or simplicity, as emotional relevance may strengthen immediate engagement and willingness to use the system. Prior studies have also noted that clear structure and reduced cognitive effort are important, but meaningful content, guided feedback, and personally relevant interaction may further support engagement among older adults [9,28,29].

This role of personal relevance can be interpreted through research on autobiographical memory. Autobiographical memories serve self-related and social functions by connecting past experiences to a continuing sense of identity and to relationships with others [30]. Research on digital storytelling similarly suggests that personal media can support memory, affect, self-expression, and interpersonal connection, with many interventions involving active or co-created engagement with the material [31]. RecallLive extends this principle by automatically organizing personal photographs into memory videos and linking them with a quiz and engagement summary. Participants valued the reduced effort involved, while the sequence extended the experience beyond passive viewing by prompting responses to the presented memories. Thus, personalization in RecallLive derives not only from the use of personal photographs but also from their organization into a sequence of recall, response, and feedback.

Building on this personalized interaction, the RecallLive system contributes to the emerging field of cognitive apps for older adults by combining 3 components into one continuous process: memory organization, emotion detection, and summary generation. The integration of these components enables the system to summarize response patterns occurring during memory interaction rather than merely presenting personalized content. Related computer vision research has examined facial expressivity alongside validated measures of apathy in people with neurocognitive disorders [32]. RecallLive extends this line of work by applying facial-response analysis to personalized memory interaction, with the aim of identifying contextual patterns of engagement that may inform caregiver understanding and subsequent support. Interpretation should nevertheless remain cautious because facial movements do not always correspond directly to internal emotional states; the system’s outputs are therefore best understood as context-dependent indicators of engagement [33,34]. By focusing on patterns across time rather than single observations, the system may reduce the influence of isolated classifications and provide more stable contextual summaries.

The caregiver-facing summary extends this use of response patterns by creating a potential bridge between personal reminiscence and subsequent caregiver support. Technology-supported reminiscence may be particularly valuable when personal media provide a focal point for conversation and help others better understand the experiences of the person receiving support [35,36]. From this perspective, the summary may direct attention to memory content associated with stronger or more sustained engagement. Caregivers could use such information to prompt follow-up conversation or shared reminiscence.

The practical implementation of these functions also requires attention to privacy and ease of initial use. Privacy was therefore embedded in the system design, with all facial analyses performed on-device and no raw images stored or transmitted. This approach may reduce concerns related to surveillance and data misuse, which are commonly raised in discussions of facial recognition technologies [37]. Participants generally found the system easy to use, but some reported brief uncertainty during the first interaction. This suggests that even simple systems may benefit from short onboarding guidance, especially for users who are less familiar with mobile apps. Previous usability research supports the importance of clear instructions, simple layouts, low-burden navigation, and accessible content delivery for older adults [38-40]. Overall, the findings indicate that perceived usefulness was associated with stated intention to use, while positive engagement and usability evaluations characterized participants’ initial interaction with the system. These findings warrant further evaluation under longer-term and real-world conditions.

### Limitations

This study has several limitations. First, although phase 1 included a large sample, participants evaluated the system through a demonstration video. This may not fully reflect real interaction. Phase 2 partially addressed this by including hands-on use, but the smaller sample size limits generalization. The phase 2 sample comprised 10 of the 202 phase 1 participants. Eligibility required ownership of a compatible Android smartphone, the ability to install the APK-based app, and willingness to complete a hands-on session and interview within a 1-week window. These requirements likely restricted the pool of potential volunteers. In addition, since phase 1 participants were recruited online and phase 2 participants were drawn from this sample, participation required at least a minimum level of digital literacy and internet access. This may have introduced selection bias and may limit the generalizability of the findings to older adults with lower digital literacy or limited internet access. Second, the evaluation was conducted over a short period and did not capture long-term engagement. Future research should examine sustained interaction over time, particularly in relation to cognitive outcomes. Given the increasing prevalence of cognitive decline [1,2], longitudinal studies are necessary to assess real-world impact. Third, the emotion recognition component has known limitations; namely, facial expression data provide partial insight and should not be treated as a complete representation of emotional state [33]. Future systems may improve accuracy by incorporating additional signals such as voice or interaction patterns. Finally, future research should explore broader app contexts, including integration with caregiver systems and clinical environments. Advances in data-to-text generation may further improve interpretability and usability of system outputs [41,42].

### Conclusions

Overall, the results showed high levels of engagement and perceived usefulness, with the latter strongly associated with stated intention to use the system. By combining structured memory interaction with on-device emotional analysis, RecallLive appears to offer a usable and meaningful approach to supporting cognitive engagement among older adults, with potential for real-world application. Further evaluation through longer-term studies and clinically integrated settings is warranted.

## Supplementary material

10.2196/103986 Multimedia Appendix 1 Semistructured interview guide for phase 2 usability sessions.

10.2196/103986 Checklist 1 CHERRIES checklist.

## Abbreviations

APK: Android Package Kit
CHERRIES: Checklist for Reporting Results of Internet E-Surveys
LLM: large language model
OLS: ordinary least squares
RCSS: RecallLive Cognitive Support System
RRA: ReLive Response Analyzer
RRG: ReLive Report Generator
RVC: ReLive Visual Composer
TAM: Technology Acceptance Model
