# Supplementary material for: An AI-Assisted Cognitive Engagement Mobile App for Older Adults: Development and Mixed Methods Usability Study
Source: JMIR Form Res. 2026 Sep 10;10:e103986. doi: 10.2196/103986 (PMC13561194; doi:10.2196/103986)
Supplement: Supplementary file 2 [file formative-v10-e103986-s001.docx]

**Appendix 1. Semi-Structured Interview Guide for Phase 2**

**Ease of Use**

Can you tell me about your experience using the app. How was navigating and completing tasks?

*Follow-up:* Was there anything confusing or unclear? What made it easy or difficult?

**Engagement**

How did you feel while using the app, particularly when interacting with the photo and video features?

*Follow-up:* What made the experience enjoyable or not enjoyable? Did it make you reflect on your memories?

**Design & Accessibility**

What did you think about the app's visual design and layout?

*Follow-up:* Was anything difficult to see or visually unclear? Did anything about the layout stand out to you?

**Perceived Usefulness**

In what ways do you find this app useful for supporting memory or daily life?

*Follow-up:* In what situations would this be helpful? Who do you think this app is most suited for?

**Intention / Recommendation**

How likely would you be to use this app in the future or share it with others? Would you consider using this app regularly or recommending it to others? Why or why not?

*Follow-up:* What factors would influence your decision? What might encourage or discourage you from using it?

**Comparison (Demonstration vs Actual Use)**

How did this firsthand experience compare to when you saw the demonstration earlier?

**Additional Open-Ended Questions**

What did you like most about the app?

What was confusing or difficult to use?

What improvements would you suggest?
